# Prevalence and predictors of dual antiplatelet therapy prolongation beyond one year in patients with acute coronary syndrome

**DOI:** 10.1371/journal.pone.0186961

**Published:** 2017-10-23

**Authors:** Giuseppe Patti, Ilaria Cavallari, Emilia Antonucci, Paolo Calabrò, Plinio Cirillo, Paolo Gresele, Gualtiero Palareti, Vittorio Pengo, Pasquale Pignatelli, Elisabetta Ricottini, Rossella Marcucci

**Affiliations:** 1 Campus Bio-Medico University of Rome, Rome, Italy; 2 Arianna Anticoagulazione Foundation, Bologna, Italy; 3 Division of Cardiology, Monaldi Hospital and "Luigi Vanvitelli" University of Campania, Naples, Italy; 4 Department of Advanced Biomedical Sciences, School of Medicine, "Federico II" University, Naples, Italy; 5 Department of Medicine, Division of Internal and Cardiovascular Medicine, University of Perugia, Perugia, Italy; 6 Department of Cardiothoracic and Vascular Sciences, University Hospital of Padua, Padua, Italy; 7 Department of Internal Medicine and Medical Specialties, La Sapienza University of Rome, Rome, Italy; 8 Department of Experimental and Clinical Medicine, Center for Atherothrombotic diseases, University of Florence, Florence, Italy; Medizinische Hochschule Hannover, GERMANY

## Abstract

There are limited real-world data on prevalence and predictors of dual antiplatelet therapy (DAPT) prolongation beyond one year after acute coronary syndrome (ACS). We have explored such issue in the START ANTIPLATELET Registry, which is a prospective, observational, multicenter, Italian registry performed in seven Italian cardiology institutions including patients admitted for ACS and followed up to one year. Out of a total population of 840 ACS patients, 596 patients had completed 12-month follow-up being on DAPT. Decision to prolong DAPT beyond one year was taken in 79 patients (13%), whereas in 517 patients DAPT was stopped. The strongest predictors of DAPT continuation were a new cardiovascular events after the index admission event (OR 3.3, 95% CI 1.4–7.7), no bleeding complications (OR 3.2, 95% CI 1.2–8.3) and no anemia during one-year follow-up (OR 2.6, 95% CI 1.1–5.9); other independent predictors were renal failure (OR 2.5, 95% CI 1.3–5.0) and peripheral artery disease (OR 1.8, 95% CI 1.1–3.0). The choice of DAPT prolongation was not correlated with younger ager, presence of diabetes mellitus, coronary angioplasty as initial treatment strategy or type of implanted stent (drug-eluting vs bare metal). In conclusion, this study provides a real-world snapshot on the factors influencing the option to continue DAPT beyond one year after ACS; a low bleeding risk seems to influence the choice to prolong DAPT more than a high ischemic risk.

## Introduction

Current guidelines on myocardial infarction (MI) and non-ST-segment elevation acute coronary syndromes (NSTE-ACS) recommend as routine strategy the use of dual antiplatelet therapy (DAPT, aspirin plus a P2Y12 inhibitor) up to one year after the index event and then to continue with a single-drug approach, usually aspirin.[1–3] However, large-sized registries have shown that at least 20% of patients who are event-free at one year post-MI and receive a single antiplatelet treatment will suffer a new cardiovascular event within five years.[4,5] On the other hand, sub-analyses from controlled randomized trials had suggested that DAPT prolongation beyond one year after ACS is associated with reduction of ischemic cardiovascular complications;[6,7] more recently, the randomized PEGASUS and DAPT trials have demonstrated a decrease of major adverse cardiovascular events (MACE) with prolonged DAPT after MI or coronary stenting, respectively, compared to aspirin alone, at the price of a significant increase of non-fatal bleeding.[8,9] Thus, at one year after ACS it appears crucial to carefully consider and balance on an individual basis the ischemic and bleeding risks and tailor a long-term antithrombotic strategy approach accordingly. Of note, the 2015 Guidelines of the European Society of Cardiology on NSTE-ACS indicate the possibility to use in selected patients a P2Y12 inhibitor in addition to aspirin beyond one year with class of recommendation IIb and level of evidence B.[1]

To date, there are limited real-world data on the proportion of patients for whom the prolongation of DAPT beyond one year after ACS is deemed favorable in terms of net clinical benefit; moreover, no previous study has specifically evaluated independent predictors of DAPT prolongation in this setting. Thus, we have explored this issue in the multicenter, Italian START ANTIPLATELET Registry.

## Materials and methods

START ANTIPLATELET is a prospective, real-world registry performed in seven Italian cardiology institutions on patients admitted for ACS. In this paper we present data on the first 840 patients who have completed 1-year follow-up by January 31, 2017. Inclusion criteria were: age ≥18 years; written informed consent for study participation; admission for ACS (either STEMI or NSTE-ACS). To reduce selection bias, no explicit exclusion criteria were present; moreover, two specific and fixed working days in the week (for example Tuesday and Friday) were chosen at each site and all consecutive patients with ACS admitted in those days were enrolled.

The study design consisted of a clinical evaluation at the time of hospital stay (baseline visit), at six-month and 1-year follow-up. Demographic data, clinical characteristics, risk factors and treatment modalities were collected at baseline; the occurrence of adverse events, both cardiovascular events and bleeding complications, was recorded at the 1-year evaluation, as well as the type of therapy given during follow-up, drug-related side effects, duration and compliance to antithrombotic treatments. Only documented adverse events were considered relevant, as defined in current guidelines and with the date of any event being after the baseline visit. Individual data were entered into an electronic case report form including various plausibility checks for the considered variables.

START-ANTIPLATELET is a branch of the START registry (Survey on anTicoagulated pAtients RegisTer, NCT02219984),[10] promoted by the Arianna Anticoagulazione Foundation, Bologna. The registry was investigators-driven, non-sponsored and was approved by the Ethic Committee of each participating institution (Campus Bio-Medico University of Rome; Monaldi Hospital and "Luigi Vanvitelli" University of Campania; "Federico II" University of Naples; University of Perugia; University Hospital of Padua; La Sapienza University of Rome; University of Florence).

### Definitions and endpoints

For the purpose of this analysis, we have included only patients receiving DAPT throughout the 1-year follow-up and we have considered separately those patients according to the decision of the treating cardiologist to continue or not DAPT beyond one year. Aim was to describe independent predictors of DAPT prolongation beyond one year after ACS in a real-world setting. The study was performed before ticagrelor being licensed in Italy for clinical use beyond one year in patients with MI; therefore, DAPT continuation was performed with clopidogrel 75 mg/day in all patients. MACE were defined as cardiovascular death, myocardial infarction or stroke. Major bleeding was defined according to the Thrombolysis in Myocardial Infarction (TIMI) classification, as intracranial bleeding or clinically overt bleeding associated with a decrease in hemoglobin of more than 5 g/dL.

### Statistics

Categorical variables are expressed as number (percentage). Continuous variables are indicated as median (interquartile range), unless otherwise specified. Continuous variables were compared by t-test for normally distributed values (as assessed by Kolmogorov-Smirnov test), otherwise the Mann-Whitney U-test was applied. We investigated clinical characteristics being independent predictors of DAPT continuation beyond one year by logistic regression: each of the variables indicated in Table 1 was first evaluated in a univariate model, and only those variables with P value <0.15 were then entered into the final model of multivariable logistic regression analysis. Odds ratios (OR), 95% confidence interval (CI) and corresponding P values are presented. All calculations were performed by the SPSS 12.0 software and P values <0.05 (two-tailed) were considered significant.

**Table 1:** Demographic and clinical characteristics.

	No DAPT prolongation (N = 517)	DAPT prolongation (N = 79)	P value
Age (years)	68 (59;77)	70 (61;80)	0.53
Age >75 years	161 (31)	30 (38)	0.28
Female gender	117 (23)	19 (24)	0.89
Clinical presentation			0.09
STEMI	272 (53)	33 (42)	
NSTE-ACS	245 (47)	46 (58)	
Current cigarette smoking	261 (51)	37 (47)	0.63
Systemic hypertension	372 (72)	55 (70)	0.77
Diabetes mellitus	141 (27)	18 (23)	0.48
Dyslipidaemia	259 (50)	42 (53)	0.70
Previous MI	100 (19)	24 (30)	0.036
Previous PCI	87 (17)	27 (34)	0.001
Previous major bleeding	13 (3)	2 (3)	0.71
Previous TIA/stroke	29 (6)	3 (4)	0.69
Peripheral artery disease	106 (21)	24 (30)	0.07
Concomitant atrial fibrillation	39 (8)	4 (5)	0.58
LVEF ≤40%	107 (21)	19 (24)	0.60
BMI <18 kg/m^2^	2 (0.4)	0	0.62
BMI ≥ 30 kg/m^2^	100 (19)	13 (17)	0.65
Anemia*	88 (17)	8 (10)	0.17
Platelets <100.000/mm^3^	6 (1)	3 (4)	0.20
Creatinine clearance <50 mL/min	62 812)	16 (20)	0.07
Therapy for index event			
Medical therapy	22 (4)	4 (5)	0.98
CABG	6 (1)	0	0.72
PCI	489 (95)	75 (95)	0.89
PCI with stent	463 (90)	72 (91)	0.82
PCI with DES	406 (79)	66 (84)	0.38
Antithrombotic therapy up to 1-year			
Aspirin	517 (100)	79 (100)	-
Clopidogrel	145 (28)	39 (49)	<0.001
Ticagrelor	265 (51)	28 (35)	<0.001
Prasugrel	107 (21)	12 (16)	0.32
Triple therapy	-	-	
TIMI risk score	2 (1;3)	3 (2;4)	0.34
PRECISE-DAPT risk score	14 (6;26)	18 (6–29)	0.13
Any bleeding up to one year	87 (17)	5 (6)	0.025
Thrombotic CV event up to one year	23 (4)	9 (11)	0.022
Number of drugs at one year follow-up	5 (4;6)	5 (4;6)	0.14

Values are expressed as median (interquartile range) or n. (%).

BMI = Body mass index; CABG = Coronary artery bypass graft; CV = Cardiovascular; DAPT = Dual antiplatelet therapy; DES = drug-eluting stent; LVEF = Left ventricular ejection fraction; NSTE-ACS = Non ST-segment elevation acute coronary syndrome; MI = Myocardial infarction; PCI = Percutaneous coronary intervention; STEMI = ST-segment elevation myocardial infarction; TIA = Transient ischemic attack.

*Defined as Haemoglobin <12.5 g/dL if male, <11.5 g/dL if female.

## Results

Out of a total population of 840 ACS patients with complete follow-up, 596 patients had continued DAPT up to 12 months; at 1-year evaluation, in 79 (13%) patients the treating cardiologist decided to prolong DAPT, whereas in 517 patients (87%) DAPT was stopped.

Table 1 shows demographic and clinical characteristics of patients continuing or not continuing DAPT beyond one year. Patients who prolonged DAPT had a significantly higher prevalence of previous percutaneous coronary intervention (PCI) and MI prior to the index event compared to those who did not (34% vs 17% and 30% vs 19%, respectively), as well as more elevated use of clopidogrel, instead of the newer P2Y12 inhibitors, at baseline (49% vs 28%). The prevalence of elderly patients (age >75 years), BMI at the extremes (potentially influencing the perceived bleeding risk during antiplatelet therapy), diabetes mellitus, prior transient ischemic attack (TIA)/stroke, use of PCI with stenting (vs medical therapy or bypass surgery) and penetration of drug-eluting stents (DES) were similar in the two groups, whereas there was a trend towards higher NSTE-ACS presentation, peripheral artery disease and renal failure in patients with DAPT continuation (S1 File).

**Fig 1:**
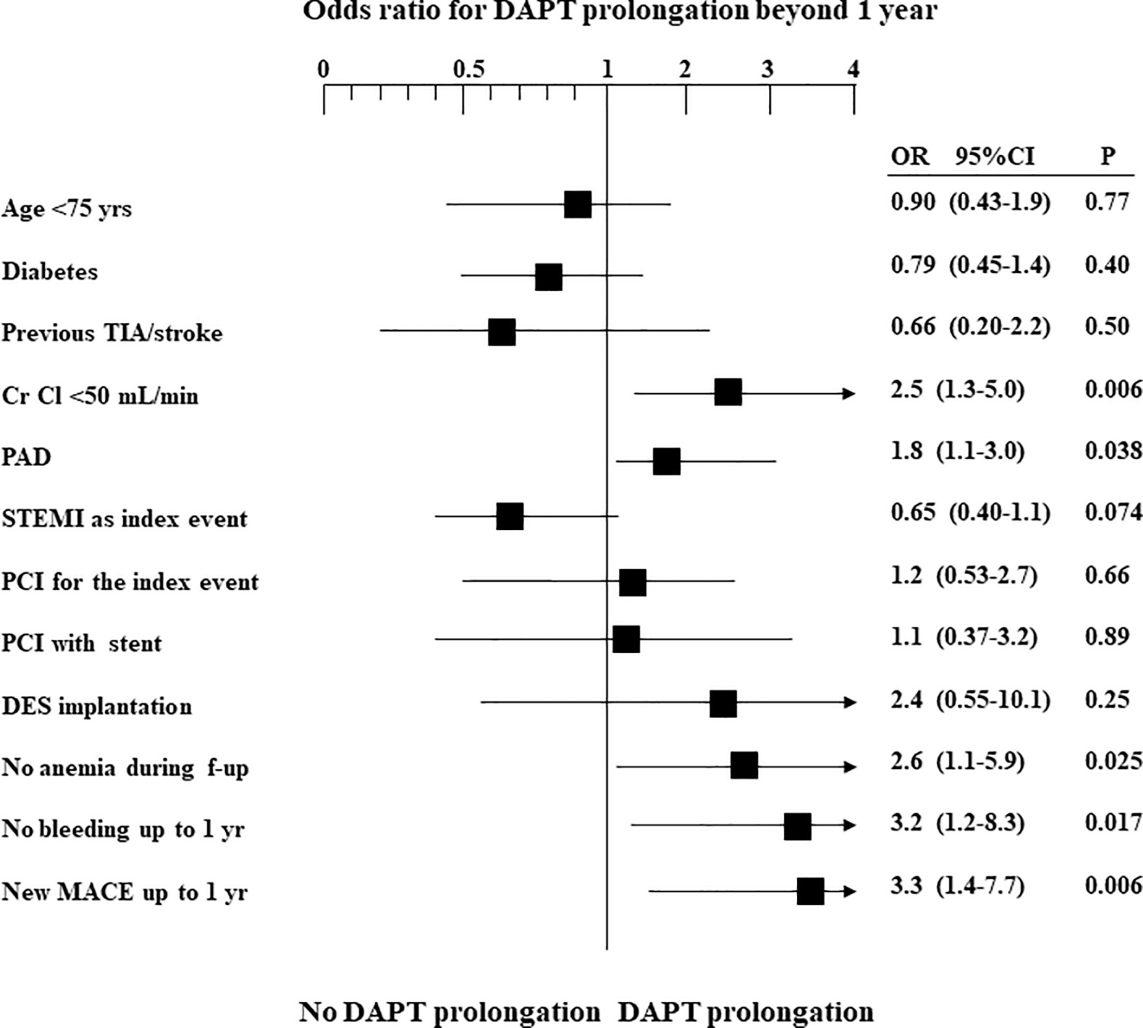
Logistic regression analysis for independent predictors of DAPT prolongation beyond one year after ACS. DAPT = Dual antiplatelet therapy; Cr Cl = Creatinine clearance; DES = Drug-eluting stent; MACE = Major adverse cardiovascular events; PAD = Peripheral artery disease; PCI = Percutaneous coronary intervention; STEMI = ST-segment elevation myocardial infarction; TIA = Transient ischemic attack.

At multivariable analysis, independent predictors of DAPT prolongation beyond one year were recurrent ischemic events (OR 3.3, 95% CI 1.4–7.7), moderate to severe renal failure (OR 2.5, 95% CI 1.3–5.0), peripheral artery disease (OR 1.8, 95% CI 1.1–3.0), no anemia (OR 2.6, 95% CI 1.1–5.9) and no bleeding event during 1-year follow-up (OR 3.2, 95% CI 1.2–8.3) (Fig 1). No relationship between younger ager, presence of diabetes mellitus, PCI as treatment strategy for the ACS, DES implantation and DAPT prolongation was observed (S1 File).

**Fig 2:**
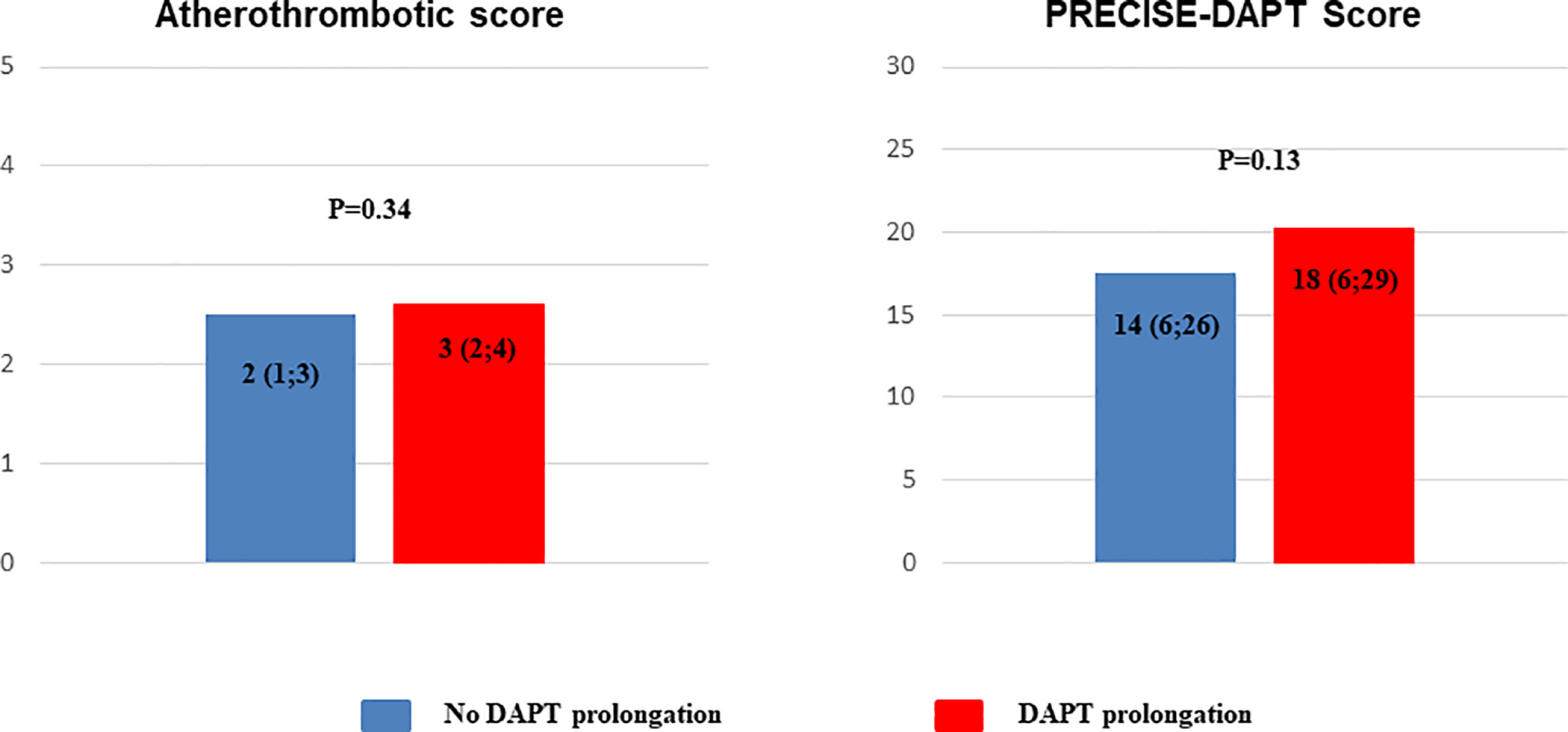
Median (interquartile range) score values in patients with and without DAPT prolongation. The atherothrombotic score derived from the TRA 2°P population indicates the risk of major adverse cardiac events, the PRECISE-DAPT score the risk of bleeding. DAPT = Dual antiplatelet therapy.

We have also assessed the ischemic and bleeding risk profile of patients continuing and not continuing DAPT by two contemporary scores, i.e. the atherothrombotic risk score derived from the TRA 2°P population [11] and the PRECISE-DAPT score for the bleeding risk;[12] interestingly, values of both scores were similar in patients with and without DAPT prolongation (Fig 2).

## Discussion

In this prospective, real-world registry we have investigated clinical variables leading to prolongation of DAPT beyond one year from ACS. We found that DAPT was prolonged in 13% of patients and independent predictors of prolongation were recurrent ischemic events, the absence of anemia or bleeding during follow-up and concomitant renal failure or peripheral artery disease. Instead, we observed no influence of lower age, diabetes mellitus or DES implantation on DAPT continuation.

Among patients with MI, observational data and post-hoc analyses of randomized trials indicated a relevant residual cardiovascular risk over the long-term in those receiving aspirin therapy alone [4–7] and suggested a clustering of adverse ischemic events in the first months after DAPT discontinuation.[13] Therefore, in the last years a mounting evidence on the clinical benefit of DAPT prolongation beyond one year after ACS has been made available. In particular, the DAPT trial compared 30 versus 12 months of DAPT with clopidogrel or prasugrel plus aspirin after coronary stenting;[9] in this study the reduction in MACE for continued thienopyridine was greater in the subgroup with MI as index event, but at the cost of increased bleeding.[14] The other randomized investigation on the topic is the PEGASUS trial (8), in which patients with a history of MI 1–3 years prior were enrolled and prolonged use of ticagrelor at two different doses (60 mg and 90 mg twice daily), given on top of aspirin therapy, was associated with significant decrease of MACE versus placebo. Both ticagrelor arms showed higher incidence of major bleeding complications, but no increase in fatal or intracranial bleeding. Given its more favorable benefit/risk ratio, 60 mg twice daily has been considered the dose of choice of ticagrelor for DAPT prolongation after an acute coronary event and represents the dose licensed in the Unites States and in various European countries. Of note, a recent meta-analysis showed a 26% and 16% risk reduction of MACE and cardiovascular mortality, respectively, with DAPT continuation at the price of >2-fold higher risk of non-fatal major bleeding, especially when the newer, more potent P2Y12 antagonists were used.[15] Thus, a careful evaluation of both bleeding and ischemic risk is mandatory in patients on DAPT completing 1-year follow-up after ACS, when DAPT may be prolonged if the estimated risk of ischemic events on aspirin alone overcomes the expected DAPT-related bleeding propensity.

In the START ANTIPLATELET registry 13% of patients continued DAPT beyond one year; powerful predictors of DAPT continuation were the absence of anemia or bleeding events during 1-year follow-up (OR 2.6 and 3.2, respectively). Thus, in a real-world setting the concern of haemorrhagic complications related to a more aggressive antiplatelet treatment is predominant for choosing the optimal antithrombotic strategy at one year after ACS; accordingly, the perception of a low bleeding risk, characterized by the absence of anemia and no history of bleeding, identifies patients considered suitable for DAPT continuation. Notably, anemia and history of bleeding have been included in several scores stratifying the bleeding risk in patients receiving antithrombotic therapies.[12,16] However, in our study the clinical perception of a low bleeding risk, resulting in DAPT continuation beyond one year, did not translate into a lower bleeding risk profile as assessed by the PRECISE-DAPT score. Furthermore, patients with prior stroke or TIA are known to be at higher risk of bleeding complications during antithrombotic therapies; we observed a lower rate of DAPT continuation in patients with prior stroke/TIA (9% vs 14%), not statistically significant due to the low number of patients.

In our study markers of higher cardiovascular risk leading to DAPT prolongation were concomitant peripheral artery disease and chronic renal failure. Previous data showed that the association of coronary and peripheral artery disease identifies patients with poorer cardiovascular outcome,[17] and in the PEGASUS trial patients with concomitant peripheral artery disease achieved a larger net clinical benefit from use of ticagrelor.[18] In patients with coronary disease the presence of chronic renal failure increases the risk of ischemic events; of note, use of ticagrelor beyond one year was associated with greater absolute reduction of MACE in the subgroup with renal failure.[19] Although renal failure is certainly also a predictor of elevated bleeding risk, our real-world data indicate that in this setting of patients the concern of high risk of ischemic events if DAPT is not prolonged outweighs the concern of DAPT-related bleeding.

Older age (>75 years) has been demonstrated to significantly enhance the risk of bleeding related to antiplatelet therapies with the newer, more potent agents, in particular prasugrel or vorapaxar.[20–22] In START ANTIPLATELET, where, if considered indicated, DAPT prolongation was performed with clopidogrel as P2Y12 inhibitor, older age was not a deterrent for stopping DAPT at one year. Of note, diabetes mellitus was not a predictor of DAPT continuation. It is well known that diabetic patients have a higher prevalence of impaired response to clopidogrel [23,24] and subgroup analysis from the CURE trial had suggested a lower ischemic protection with aspirin plus clopidogrel vs aspirin alone in diabetic versus non-diabetic patients with ACS;[25,26] of note, in the DAPT study the clinical benefit of the association aspirin plus P2Y12 inhibitor was attenuated in patients with diabetes compared to those without.[27] All those observations may contribute to explain our results on the lack of relationship between presence of diabetes and DAPT prolongation. Moreover, we observed that DAPT continuation was irrespective of the therapeutic strategy for the index event, i.e. it was performed in patients treated with either medical therapy or PCI. This is consistent with findings of the PLATO trial,[28] indicating an elevated risk of adverse events in patients unsuitable for coronary revascularization after ACS; of note, the DAPT study showed that long-term DAPT, beyond the reduction of events related to the culprit coronary vessel (i.e. stent thrombosis or target vessel revascularization), prevented progression and destabilization of athero-thrombotic processes in the entire coronary tree.[14] Finally, in the START ANTIPLATELET registry there was not a higher prevalence of DAPT prolongation in patients receiving DES versus bare metal stents (14% vs 11%); this is consistent with recent data showing that the risk of stent thrombosis with the newer-generation DES is very low, whereas the risk of such complication after bare metal stent implantation is not negligible.[29]

At the time of the START ANTIPLATELET registry ticagrelor 60 mg was not licensed in Italy for long-term use after MI; when this formulation of ticagrelor will become available for clinical use, other features of high bleeding risk (i.e. older age) are likely to become important for the decision on DAPT prolongation beyond one year with this drug. Moreover, in the PEGASUS trial diabetic patients obtained a greater net clinical benefit from ticagrelor administration, [30] and it is possible that, unlike so far done, the presence of diabetes will become in the near future an incentive for DAPT continuation beyond one year after MI. Of note, we found that patients with DAPT prolongation had a significantly higher use of clopidogrel, instead of the newer, more potent P2Y12 inhibitors, during 1-year follow-up; therefore, in the real-world setting of our registry, a switching at one year from ticagrelor or prasugrel to clopidogrel was not frequently performed, although patients receiving these more potent agents after an ACS are usually at higher risk of ischemic events.

Risk scores, such as the score derived from the TRA 2°P population and the PRECISE-DAPT score (for ischemic and bleeding events, respectively), have been recently proposed as useful tools to predict the risk of future adverse events and guide physicians in the selection of patients who may derive a net benefit from prolonged DAPT.[11,12] However, our registry, started before the diffusion of those scores, did not show any difference in either ischemic or bleeding scores between patients who discontinued or prolonged DAPT at one year. It is possible that in the future a larger use of these scores in routine practice will implement the decision-making process regarding optimal DAPT duration after ACS.

We recognize limitations in our study. First, the study size is a relevant statistical limitation. Moreover, bias in the patients’ selection and residual confounding cannot be excluded; finally, we were not able to evaluate the predictive role for DAPT prolongation of various angiographic and procedural variables (i.e. multivessel disease, multivessel intervention, degree of coronary calcification, number of stents), because those variables were not collected.

In conclusion, START ANTIPLATELET provides a real-world snapshot about the decision of prolonging DAPT beyond one year after ACS in a European country and describe clinical factors influencing the choice of this strategy; moreover, it illustrates the relative contribution of low bleeding risk versus high ischemic risk features for DAPT continuation. According to our data, a low bleeding risk seems to weight more than a high ischemic risk in the current decision for DAPT prolongation. It will be intriguing to evaluate how the predictors of DAPT continuation will change after the introduction of 60 mg ticagrelor for long-term prevention of atherothrombotic events after MI.

## Supporting information

S1 File (XLS)
